# Imaging Reporter Strategy to Monitor Gene Activation of Microglia Polarisation States under Stimulation

**DOI:** 10.1007/s11481-018-9789-2

**Published:** 2018-05-22

**Authors:** Franziska M. Collmann, Rory Pijnenburg, Gabriele Schneider, Cordula Schäfer, Kat Folz-Donahue, Christian Kukat, Mathias Hoehn

**Affiliations:** 1 Max Planck Institute for Metabolism Research, In-vivo-NMR Laboratory, Gleueler Straße 50, D-50931 Cologne, Germany; 2 Max Planck Institute for Biology of Ageing, FACS & Imaging Core Facility, Cologne, Germany; 3 Leiden University Medical Center, Radiology, Leiden, The Netherlands; 4 PERCUROS, Enschede, The Netherlands

**Keywords:** Microglia, Reporter imaging, Bioluminescence imaging, Fluorescence imaging, Microglia polarisation

## Abstract

Microglial cells as innate immune key players have a critical and unique role in neurodegenerative disorders. They strongly interact with their microenvironment in a complex manner and react to changes by switching their phenotype and functional activation states. In order to understand the development of brain diseases, it is imperative to elucidate up- or down-regulation of genes involved in microglia polarisation in time-profile by a simple-to-use strategy. Here, we present a new imaging strategy to follow promoter activity of genes involved in microglia polarisation. We lentivirally transduced BV-2 microglia cells in culture with constructs consisting of the induced nitric oxide synthase (iNOS), Fc gamma receptor III (Fcgr3) (both resembling the pro-inflammatory M1-like phenotype) or Chitinase-like 3 (Chil3/Ym1) (resembling the anti-inflammatory M2-like phenotype) promoters and stimulated transgenic cells with potent activators for pro- or anti-inflammatory response, such as lipopolysaccharide (LPS) + interferon gamma (IFN-γ) or interleukin (IL)-4, respectively. Promoter activities upon polarisation phases were quantitatively assessed by the two imaging reporters Luc2 for bioluminescence and eGFP for fluorescence.

**Electronic supplementary material:**

The online version of this article (10.1007/s11481-018-9789-2) contains supplementary material, which is available to authorized users.

## Introduction

Microglial cells, as the innate immune key players of the brain, take on a crucial and complex role during various neurodegenerative disorders, such as stroke (Hu et al. 2012; Xiong et al. 2016), multiple sclerosis, Parkinson’s disease and Alzheimer’s disease (Cherry et al. 2014). Microglia can change their morphological and phenotypic activation state in response to their microenvironment. Potent activators, such as cytokines and chemokines have been shown to induce pro- and anti-inflammatory activation states, typically labelled with the somewhat simplified concept of M1 and M2 states, respectively (Chhor et al. 2013; Martinez and Gordon 2014; Prinz and Priller 2014). During different phases of a disease, microglia may shift their phenotypes between these polarisation states. Further, a detailed and time-dependent monitoring of this phenotype shift is required to achieve a better understanding of the role of the immune activity for the development of brain diseases. Such knowledge is sought for the development of novel therapeutic strategies based on immune modulation. Additionally, drug screening profits from an inclusion to study the drug’s interaction with the immune system already under controlled cell culture conditions, even before further in vivo experiments are performed.

We report here a novel imaging reporter-based strategy for monitoring the microglia phenotype. As a model system we performed stimulation experiments in cell culture of a single BV-2 microglia cell line that was generated by immortalisation of murine primary microglia by Blasi and colleagues in 1990 (Blasi et al. 1990). Cells were transduced with lentiviral (LV) particles consisting of the two reporter genes: The codon-optimised firefly luciferase (Luc2) and enhanced green fluorescent protein (eGFP), covalently expressed through the T2A peptide, derived from the *Thosea asigna* virus (de Felipe et al. 2006), and controlled by the M1- (induced nitric oxide synthase (iNOS), Fc gamma receptor III (Fcgr3)) or M2-like (Chitinase-like 3 (Chil3/Ym1)) promoters. In line with several reports, these three genes are relevant markers for pathology, such as stroke, parasitic infections or alveolar diseases (Hung et al. 2002; Colton 2009; Bruhns 2012; Hu et al. 2012; Kawahara et al. 2012; Garry et al. 2015; Chen et al. 2017). Furthermore, transcriptome analyses of macrophages confirmed iNOS and Ym1 as relevant M1- and M2-like markers, respectively (Jablonski et al. 2015). As representative activators to induce the M1-like and M2-like phenotypes, we applied lipopolysaccharide (LPS), derived from the gram-negative bacterial cell wall, together with the T helper cell type 1 cytokine interferon gamma (IFN-γ) and the T helper cell type 2 regulator, interleukin (IL)-4, respectively.

We demonstrate that our approach allows easy and rapid generation of in vitro stimulation results, in good agreement with the conventionally used but time-consuming Western blot (WB) method. But different to WB, our reporter strategy focuses on monitoring promoter activity, while it is insensitive to any post-transcriptional or post-translational modifications.

## Materials and Methods

### Cloning

All expression constructs were generated from the pCDH-EF1α-Luc2-T2A-eGFP construct (derived from pCDH-EF1α-MCS-T2A-copGFP (cat. no. CD521A-1, System Biosciences, Palo Alto, CA, USA)) (details are found in (Tennstaedt et al. 2015)) by replacing the elongation factor 1 alpha (EF1α) promoter with the iNOS, Fcgr3 or Chil3/Ym1 promoter. Prior to cloning of the promoters, the copGFP sequence was replaced by the eGFP sequence, derived from pmeGFP-1, a gift from Benjamin Glick (Addgene plasmid # 36409). pGL2-NOS2Promoter-Luciferase (Addgene plasmid #19296) was a gift from Charles Lowenstein (Lowenstein et al. 1993). Ym1 (MPRM18306-PG04) and Fcgr3 (MPRM15197-PG04) plasmid DNA were obtained from GeneCopoeia (Rockville, MD, USA). Promoter sequences were amplified with specific primers designed with corresponding restriction sites (Suppl. Table 1) by PCR. The backbone construct was linearised with the restriction sites flanking the EF1α promoter and the amplicon ligated. Gene cards of the original constructs as well as of the backbone and of the generated constructs are shown in the supplements (Suppl. Fig. 1**–**7). Gene maps were made in the free software SnapGene Viewer, while sequencing primers, as well as PCR primers were created with the software VectorNTI Advance (TM) (Version 11.0, Life Technologies, Carlsbad, CA, USA).

### Cell Lines

**Fig. 1:**
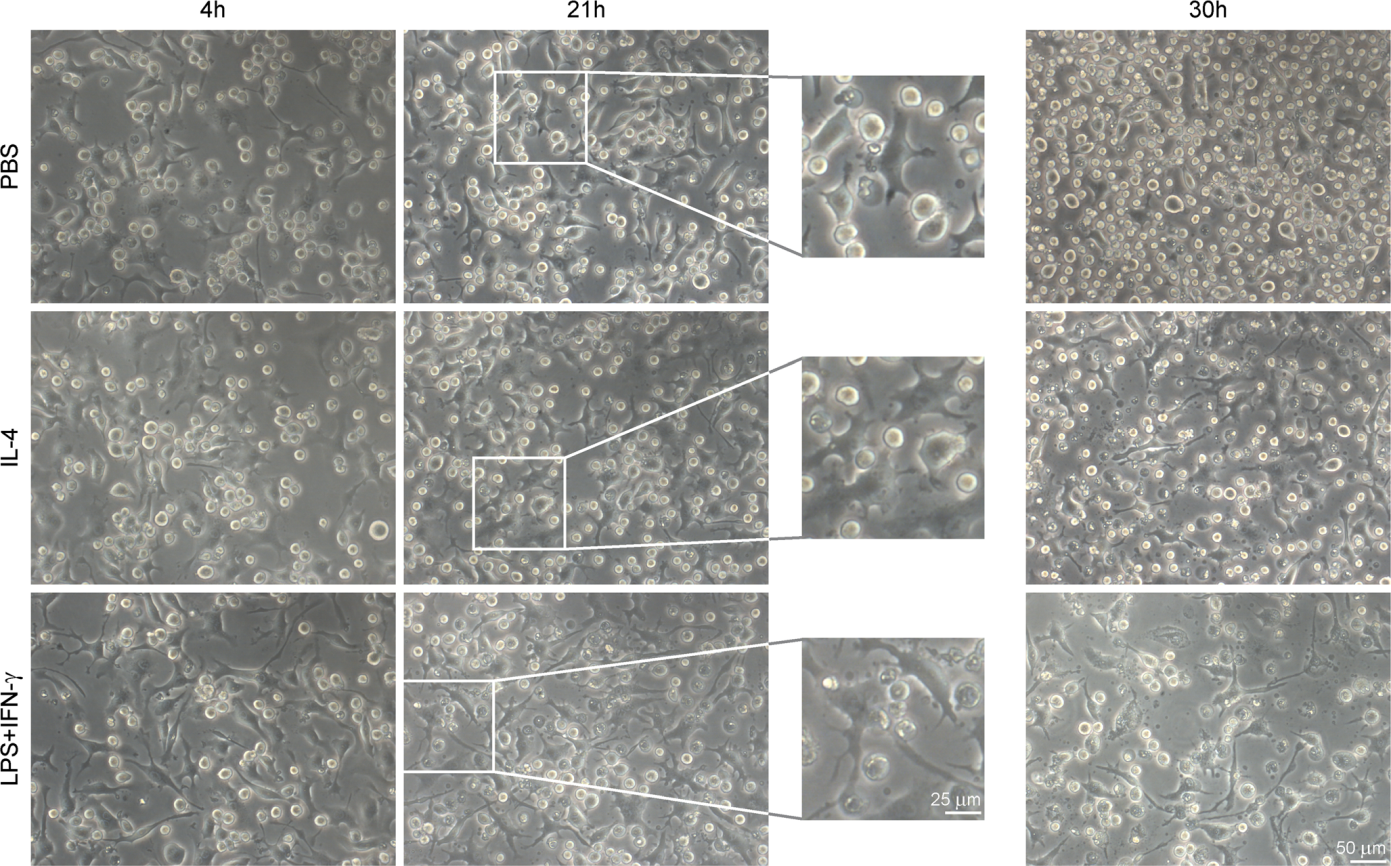
**Cell morphology of BV-2 microglia after stimulation with IL-4 or LPS + IFN-γ.** Already after 4 h of LPS + IFN-γ stimulation, cells revealed longer extensions and bigger cell bodies compared with cells stimulated with IL-4 (or PBS). After 21 and 30 h of LPS + IFN-γ stimulation, cell morphology was still changed. 20X magnification images with respective zoomed images of the 21 h time point (centre)

BV-2 cells (accession no. ATL03001, Cell bank Interlab Cell Line Collection, Italy) were cultivated in Roswell Park Memorial Institute (RPMI) medium (RPMI 1640 GlutaMAX Supplement, cat. no. 61870010, Life Technologies, Carlsbad, CA, USA), supplemented with 10% of heat-inactivated (h.i.) fetal bovine serum (FBS, cat. no. 10500–064, Thermo Fisher Scientific, Waltham, MA, USA) and 1% of penicillin/streptomycin (P/S; 10,000 U/ml, Life Technologies) (referred to as RPMI++)). Cells were passaged every 3–4 days with 0.9 × 10^5^ cells seeded in 10 cm culture dishes: Suspension cells in medium were transferred to a falcon tube and attached cells were washed and detached by flushing up and down using Dulbecco’s phosphate-buffered saline without calcium and magnesium (PBS--, cat. no. 14190169, Life Technologies). Detached cells were mixed together with suspension cells and centrifuged at 180 x g for 4 min (min). Medium was removed and cells re-suspended in the appropriate volume of fresh RPMI++ medium. Cells were counted using a Countess Automated Cell Counter (Invitrogen, Carlsbad, CA, USA). Cell morphology was inspected under a Zeiss Axiovert 40 CFL microscope (Carl Zeiss AG, Oberkochen, Germany) and images shown in Fig. 1 were taken with a Leica DFC300 FX camera (Leica, Wetzlar, Germany) using the Leica Application Suite V4.3 software (Leica).

293TN cells (cat. No. LV900A-1-GVO-SBI, Biocat GmbH, Heidelberg, Germany) were cultivated in Dulbecco’s Modified Eagle’s Medium (DMEM high glucose, GlutaMAX, pyruvate, cat. no. 31966047, Thermo Fisher Scientific), 10% h.i. FBS and 1% P/S (referred to as DMEM++). Cells were split in a ratio of 1:10 or 1:20 every 3–4 days until cells reached confluency of 80%. Medium was removed and cells were washed in PBS--, followed by trypsinisation (Trypsin-EDTA 0.05%, Thermo Fisher Scientific) for 3 min at 37 °C. Digestion was stopped by adding the threefold volume of DMEM++. Cells were centrifuged at 120 x g for 3 min and re-suspended in the appropriate volume of fresh DMEM++.

### Virus Production

Expression constructs were packed into pseudo-lentiviral particles by co-transfection with plasmids of the pPACKH1 HIV Lentiviral Packaging Kit (Biocat GmbH). 4.3 × 10^6^ 293TN cells/T75 flask were seeded in DMEM supplemented with 10% h.i. FBS. The next day, when cells reached 70–90% confluency, they were transfected with 3 μg of DNA and 10 μg of the pPACKH1 plasmid mix in Opti-Mem (cat. no. 11058021, Thermo Fisher Scientific) using Lipofectamine 3000 Transfection Reagent (Thermo Fisher Scientific). The mixture was incubated for 5 min at room temperature (RT), and the DNA-lipid complex added to the cells covered with 9 ml of Opti-Mem. After 12–15 h (h) incubation at 37 °C and 5% CO_2_, transfection medium was removed and 10 ml of fresh Opti-Mem added. LV particles were harvested on ice on the second and third day after transfection. After the first harvest, LV particles were stored at 4 °C overnight (ON), 10 ml of fresh Opti-Mem added, and the next day, viral particles mixed with harvest of the second day. Before concentration, cells were centrifuged at 240 x g for 10 min at 4 °C and then filtered through a 0.45 μm cellulose acetate filter (VWR International GmbH, Langenfeld, Germany). Unconcentrated virus was stored at −80 °C. LV particle suspension was transferred to ultra-clear tubes (cat. no. 344058, Beckman Coulter, Krefeld, Germany), and particles ultracentrifuged in a 20% sucrose (50,389, ≥99.5%, Sigma-Aldrich, St. Louis, MO, USA) cushion (in PBS--) (Beckman Coulter, Sw32-Ti swing-out bucket rotor) at 30,000 rpm for 2 h 20 min at 4 °C. Supernatant was discarded and the LV pellet dissolved in Hanks’ Balanced Salt solution (HBSS) (cat. no. 14170–070, Thermo Fisher Scientific) during slight shaking ON at 4 °C in appropriate volume to aim for a 500-fold concentration. The next day, the LV pellet was dissolved by very gentle up- and down-pipetting, and LV particle solution was stored in 10 μl aliquots at −80 °C.

### Titer Test and Transduction

Titers were examined by using the HIV-1 p24 Antigen ELISA 2.0 kit (cat. no. 0801008, ZeptoMetrix Corporation, Buffalo, NY, USA). In short, various dilutions of the viral test sample ranging from 1:200,000 to 1:5,000,000, and the HIV-1 p24 Antigen Standard probe ranging from 0 to 125 pg/ml, were prepared in Assay Diluent in duplicate. 200 μl of dilutions were added to 96-well plates coated with an antibody specific for the p24 gag protein and incubated ON at 37 °C. The next day, HRP-conjugated HIV-1 p24 Detector Antibody was incubated for 1 h at 37 °C, and then substrate added for 30 min at RT. The reaction was stopped and optical density (OD) was immediately measured at 405 nm using a Sunrise plate reader (TECAN Group Ltd., Männedorf, Switzerland). Based on the resulting standard OD curve, titers were determined in the excel software.

Cells were seeded in 100 μl medium in 96-well plates, and the next day, medium was replaced with fresh medium, including 8 μg/ml of Polybrene (Sigma-Aldrich), and 1 μl of the respective concentrated LV solution was added (titer of concentrated LV solution ranged between 1.2–3.2 × 10^7^ pg/ml). The next day, medium was replaced with fresh medium. Upon confluency of about 80%, cells were transferred to dishes with according surface areas.

### Fluorescence-Activated Cell Sorting (FACS)

After five passages, transduced cells were sorted based on their eGFP expression levels using BD FACSAria Fusion (high eGFP sorted iNOS cells) and BD FACSAria II(I)u sorters (all other cell lines; BD Biosciences, San Jose, CA, USA) (medium levels between 10^~2.2^–10^~3.7^ for EF1α cells, 10^~2.5–^10^~3.3^ for remaining cells; high levels between 10^~3.8–^10^~5^ for EF1α cells, 10^~3.4–^10^~5^ for remaining cells) (Suppl. Fig. 8). BV-2 wild type (wt) cells served as background control in each experiment. Images were post-processed in the FlowJo software Version 10.0.7 (FlowJo LLC, Ashland, OR, USA).

### Stimulation Assays

Three or four days before cells were seeded for stimulation, RPMI medium was reduced to 5% FBS h.i. without P/S. One day before stimulation, cells were seeded in plain medium in the absence of FBS. For stimulation, half of the media was removed and replaced by RPMI without FBS containing stimulants in their final concentration.. In all stimulation experiments, cells were stimulated with 100 ng/ml of LPS (from *Escherichia coli* 055:B5 L6529, Sigma-Aldrich) + 20 ng/ml of IFN-γ (cat. no. 315–05, PeproTech Inc., Rocky Hill, NJ, USA) or with 20 ng/ml of IL-4 (cat. no. 214–14, PeproTech). Supplementation with 10 μl of PBS-- served as control. Stimulation was stopped after 4, 21, or 30 h for Western Blot, or after 4, 12 h (and additionally, 24 h for BV-2 Ym1-Luc2-T2A-eGFP) for plate reader analysis.

### Western Blot

For Western Blot analysis, 1.5 × 10^6^ BV-2 cells were seeded in 4 ml plain RPMI in 6 cm culture dishes and stimulated the next day. For details on cell lysis and Western blotting refer to (Tennstaedt et al. 2015). 22 or 30 μg of wt sample and 10 μg of EF1α-Luc2-T2A-eGFP transgenic samples were loaded onto 4–20% Serva TG PRiME gels (cat. no. 43280.01, Serva Electrophoresis GmbH, Heidelberg, Germany). A list of applied antibodies can be found in Suppl. Table 2. Protein levels were determined with the software ImageJ (1.46r, Version 1.6.0, Wayne Rasband, National Institutes of Health, Bethesda, MD, USA). A region of interest (ROI) of the same size was drawn and integrated density (Intden) values after background subtraction (five random ROIs) were normalised to Intden values of actin as housekeeping protein.

### Reporter Imaging

Shortly before stimulation was stopped, cell morphology and eGFP expression were monitored under an epifluorescent microscope (BZ9000 (Biorevo), Keyence, Osaka, Japan) and images taken. Cells were shortly washed in PBS--, transferred to a 50 ml falcon tube and centrifuged at 1000 rpm for 4 min. After re-suspension in PBS-- and counting, the volume was adjusted to the necessary cell number and cells transferred to a 96-well plate (cat. no. 38840, Berthold Technologies GmbH & Co. KG, Bad Wildbad, Germany). In *n* = 3 independent experiments, 2 × 10^5^ cells were seeded in 90 μl PBS-- in each well (*n* = 6 in each experiment) to measure eGFP fluorescence and Luc2 bioluminescence reporter levels using an LB 943 Mithras^2^ plate reader (Berthold Technologies GmbH & Co. KG) running on MikroWin software (Version 5.9, Mikrotek Laborsysteme GmbH, Overath, Germany). First, eGFP levels as relative fluorescence units (RFU) were quantified at 470 nm excitation and 510 nm emission wavelength, with a counting time of 0.1 s. Thereafter, the integral reagent injector was primed with the substrate D-Luciferin (D-Luc potassium salt, 99%, BC219, Synchem UG & Co. KG, Felsberg, Germany; stock 10 mM, final concentration 1 mM) and Luc2 levels were measured as relative light units (RLU) with the following settings: slow shaking for 5 s, 300 s delay, counting time 2 s.

In each experiment BV-2 wt cells served as control and were treated in the same way as transgenic cells. For each stimulation condition, the RFU and RLU values of wt cells were subtracted as background from the respective values of the transgenic cells. Values of PBS treated cells were set to 100, and in relation to that, the ratios of IL-4 and LPS + IFN-γ values were calculated as relative changes.

### Statistical Analysis

RLU and RFU values were statistically tested by two-way analysis of variance (ANOVA), followed by Bonferroni post hoc tests using the software IBM SPSS Statistics (Version 22.0, IBM, Armonk, NY, USA). To compare reporter activity between 4 and 12 h of LPS + IFN-γ stimulation experiments, significance was tested without any post hoc test.

## Results

### Pro- and Anti-Inflammatory Stimulation Profiles of BV-2 Microglia

We have first examined vitality of all transduced and naïve cells before and after stimulation conditions. Vitality levels of transduced cells, independent of selected promoter, are in full agreement with the corresponding wild type cells, i.e. transduction was found to have no influence on cell vitality. Further, stimulation dependent vitality levels of both, wt and transgenic cells, are not significantly different from the unstimulated cells. Exception was only the LPS + INFγ stimulation for duration of 24 h which showed an equivalent, significant vitality reduction for both, wt and transgenic cells. The only difference between wt and transgenic stimulated cells was found for 12 h LPS + INFγ stimulation of the BV-iNOS cells which led to a slight but significant reduction (Suppl. Fig. 9).

Then, BV-2 microglial cells were stimulated for 4, 21, or 30 h either with IL-4 or LPS + IFN-γ, and the time-dependent cell morphological changes were observed (Fig. 1). IL-4 shifts the microglia into an M2-like anti-inflammatory phenotype, while LPS + IFN-γ was used to induce the M1-like pro-inflammatory phenotype. Already 4 h after LPS + IFN-γ stimulation, cells revealed longer protrusions compared with cells that were stimulated with IL-4 (or PBS--) (Fig. 1, left column). Upon LPS + IFN-γ induced polarisation, protrusions appeared even more distinct and longer at 21 h and were still pronounced at the 30 h time point. IL-4 stimulated cells were phenotypically indistinguishable from PBS treated cells, and due to heterogeneity of the cell population, differences in cell morphology were not clearly visible at any time point.

**Fig. 2:**
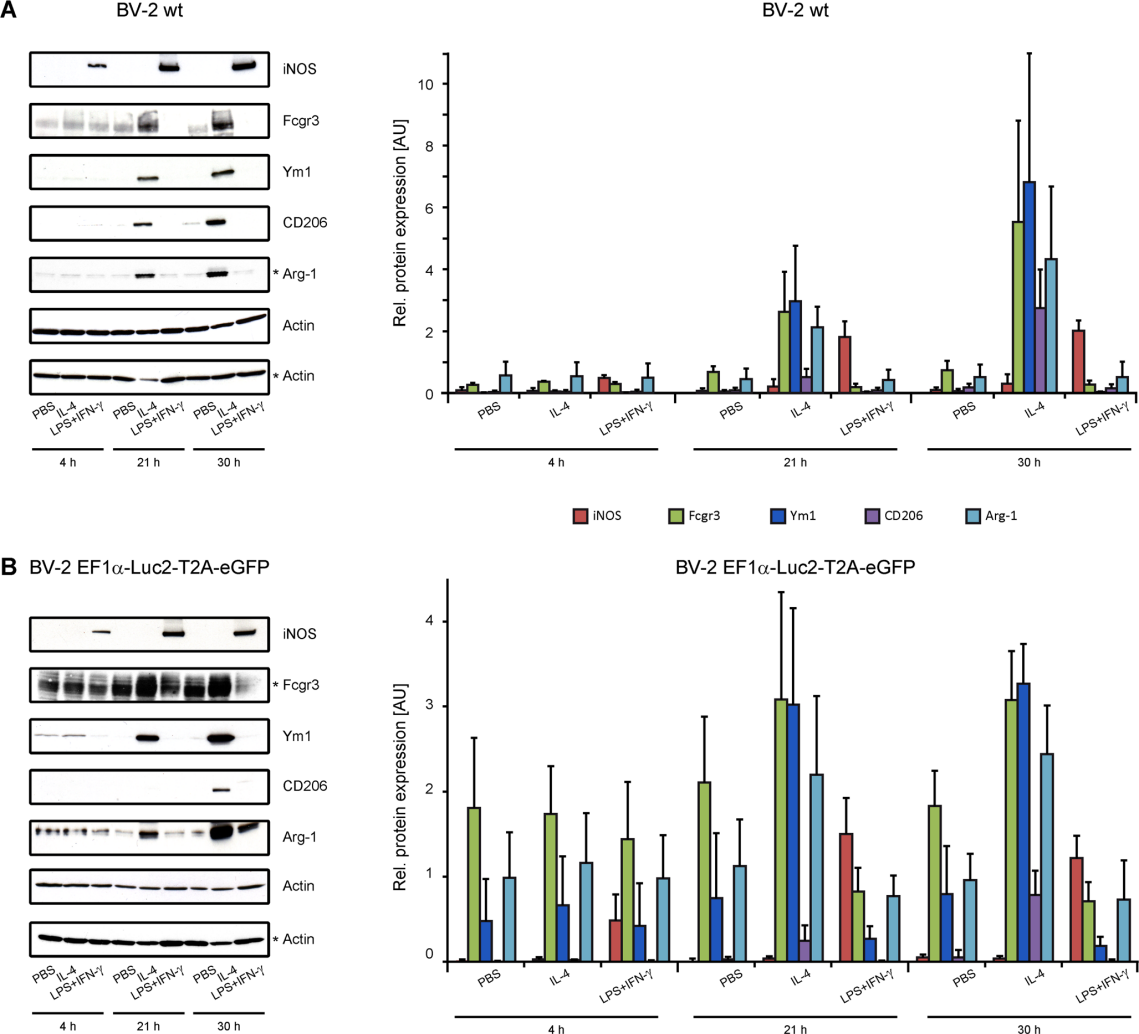
**Western Blot analysis of BV-2 cells after 4 h, 21 h and 30 h stimulation.** Stimulation was achieved with the following concentration of the agents: 100 ng/ml LPS, 20 ng/ml IL-4/ IFN-γ, 10 μl/ml PBS. BV-2 wt and transduced cells show consistent pro- and anti-inflammatory marker profiles after stimulation. WB images on the left with corresponding quantification on the right. **a** BV-2 wt cells (*n* = 3 independent experiments); **b** Transduced BV-2 EF1α-Luc2-T2A-eGFP cells. M1-like iNOS protein levels were clearly up-regulated after LPS + IFN-γ stimulation, while Fcgr3 and M2-like Ym1, Arg-1 and CD206 levels were up-regulated upon IL-4 stimulation. Stars at the Fcgr3 band and at a second Actin band indicate that these belong to a separate experiment having its own separate Actin control level

Using Western blot analysis, expression of several well-known markers of pro- and anti-inflammatory activation states (Martinez and Gordon 2014; Jablonski et al. 2015; Orihuela et al. 2016) were quantitatively assessed (Fig. 2a). LPS + IFN-γ stimulation caused a clear up-regulation of the M1-like phenotype marker iNOS, at all time points with increasing levels at 21 and 30 h compared to 4 h stimulation length. Further, iNOS expression was completely missing at IL-4 or PBS stimulation at all time points, in agreement with the literature (MacMicking et al. 1997). After 4 h of stimulation, Fcgr3 was expressed at equally low levels regardless of the stimulation treatment. IL-4 stimulation for 21 and 30 h caused an up-regulation of Fcgr3 with increased levels after 30 h of IL-4 stimulation. Even more, upon LPS + IFN-γ stimulation, Fcgr3 expression remained low at 21 h and 30 h.

Ym1, Arg-1, and CD206 levels, all markers of M2-like phenotype, were strikingly up-regulated upon IL-4 stimulation compared with PBS or LPS + IFN-γ treatment. While after 4 h of IL-4 stimulation Ym1 and CD206 were barely visible and comparable to background level (PBS stimulation), expression levels were clearly up-regulated at 21 and 30 h after stimulation. Of note, Ym1 and CD206 protein expressions were missing in LPS + IFN-γ treated cells. Though Arg-1 levels were present at all stimulation conditions at low baseline level, protein expression continuously increased upon IL-4 stimulation for 21 and 30 h. However, upon LPS + IFN-γ stimulation, Arg-1 levels did not decrease below levels of PBS treated cells at any time point.

### Evaluation of Unchanged Behaviour of Transgenic BV-2 Cells upon Stimulation

In order to control that lentiviral transduction has no effect on protein expression as reported by (Balcaitis et al. 2005), stimulation assays were repeated for BV-2 cells transduced with the EF1α-Luc2-T2A-eGFP reporter construct and markers analysed by WB (Fig. 2b). Transduced cells were selected by FACS based on middle eGFP expression, and were stimulated with the identical protocol as BV-2 wt cells in plain RPMI medium. Despite the presence of more round-shaped and less adherent cells compared to wt cells (Suppl. Fig. 10), protein patterns were analogous to those in BV-2 wt cells in terms of up- or down-regulation (Fig. 2b). We assume that transduction or FACS may cause some cell activation, as only a minor fraction of adherent cells was present in sorted transduced cells. In more detail, iNOS proteins were expressed after LPS + IFN-γ stimulation only. Also, Ym1 expression pattern were alike between transduced and wt cells in that protein levels were clearly up-regulated upon 21 and 30 h of IL-4 stimulation. Also here, Ym1 expression was entirely missing in LPS + IFN-γ stimulated transgenic cells. Likewise, in coherence with non-transduced cells, Arg-1 expression was up-regulated upon IL-4 after 21 and 30 h, while being at low baseline upon the other stimulation cues. In transduced cells, CD206 expression was up-regulated after 30 h of IL-4 stimulation, but missing at 21 h. Similar to Ym1 and to expression patterns in wt cells, CD206 protein expression was absent upon LPS + IFN-γ stimulation. Again, Fcgr3 proteins were up-regulated upon IL-4 stimulation at 30 h and much less expressed upon LPS + IFN-γ addition. In summary, we have established an appropriate stimulation protocol including cell-tolerable concentrations of stimulants, providing marker profiles of the pro- and anti-inflammatory phenotype. Further, we demonstrated that cell transduction does not modify marker expression. These findings serve as basis for the following investigation of monitoring promoter specificity and reliability of BV-2 cells transduced with the specific promoter constructs iNOS-, Fcgr3-, and Ym1-Luc2-T2A-eGFP, where stimulation response is recorded with the optical imaging reporters of bioluminescent Luc2 and fluorescent eGFP by plate reader measurements.

### Imaging Gene Reporter Activity of pro- and Anti-Inflammatory Phenotype in Transgenic BV-2 Cells

Transduced cells were sorted based on their eGFP expression (Suppl. Fig. 8) and stimulated for 4 and 12 h (iNOS and Fcgr3) and for an additional 24 h (Ym1) (*n* = 3 independent assays for each transgenic cell line). eGFP expression was inspected under the microscope for qualitative cell response to stimulation. Quantitative stimulation assessment of BV-2 cells was performed by plate reader analysis of Luc2 and eGFP signal intensity.

#### Qualitative Microscopic Analysis: eGFP Expression

**Fig. 3:**
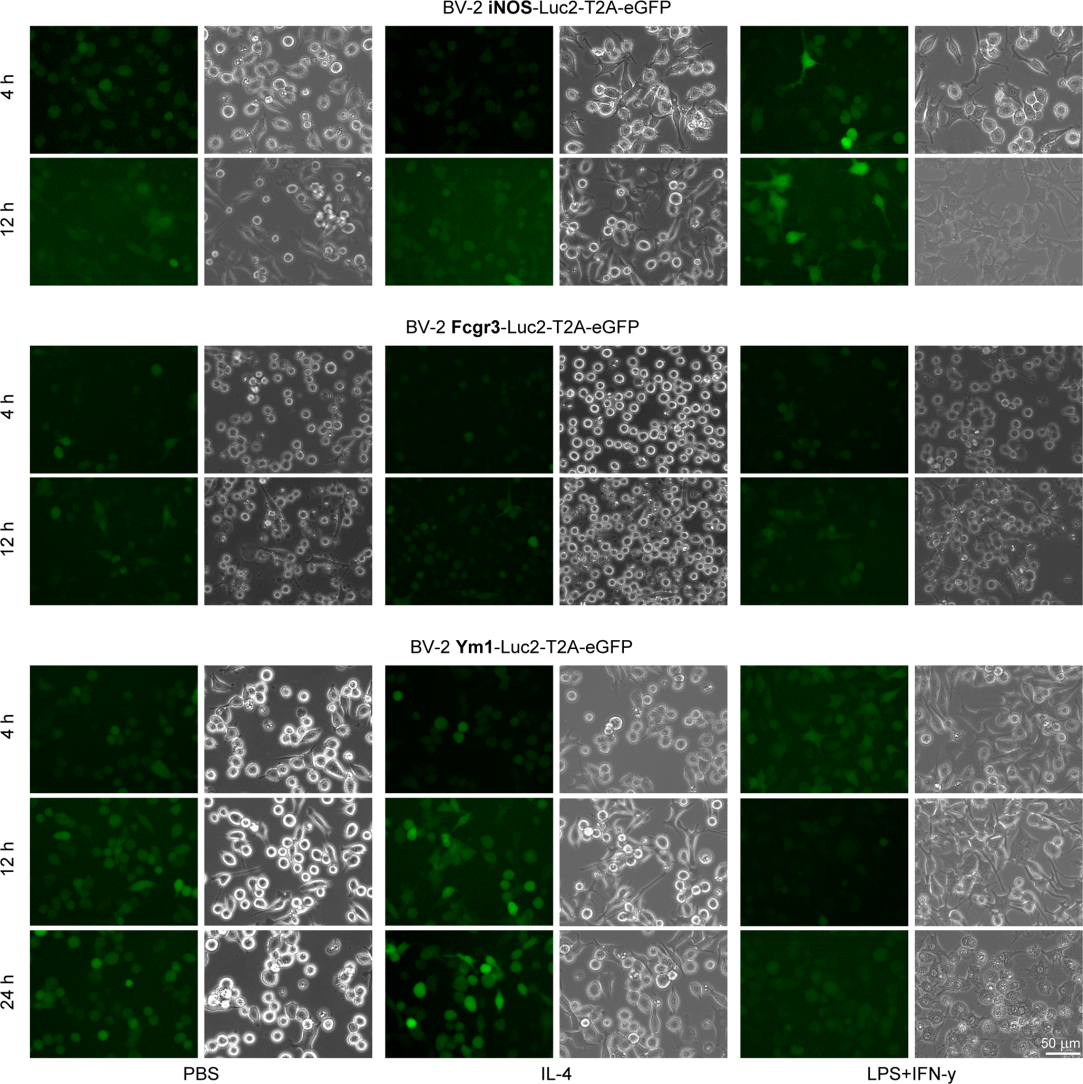
**eGFP microscopy of stimulated BV-2 cells transduced with the iNOS-, Fcgr3- and Ym1-Luc2-T2A-eGFP constructs.** Representative images of transduced cells showing the eGFP channel on the left and the corresponding phase contrast image on the right. eGFP signal increased in BV-2 iNOS cells after 4 and 12 h of LPS + IFN-γ stimulation (top). No eGFP signal change was obvious in BV-2 Fcgr3 cells (centre). In BV-2 Ym1 cells eGFP signal increased after 12 and 24 h of IL-4 stimulation (bottom). 20X magnification

Cells transduced with the iNOS-promoter construct (Fig. 3, upper set of rows) showed a strong increase of eGFP expression after LPS + IFN-γ stimulation at both time points, in comparison with PBS treated cells. This increase in fluorescence signal was missing upon IL-4 stimulation. In cells transduced with the Fcgr3 construct (Fig. 3, central set of rows), changes in eGFP signal were not clearly visible at any time point. In BV-2 cells transduced with the Ym1-promoter construct (Fig. 3, bottom set of rows), eGFP signal increased particularly after 12 and 24 h of IL-4 stimulation compared with PBS treated cells. Here, an increase of eGFP expression was missing in LPS + IFN-γ stimulated cells at all time points.

#### Quantitative Imaging Based Analysis: Luc2 Activity (RLU) and eGFP Expression (RFU)

**Fig. 4:**
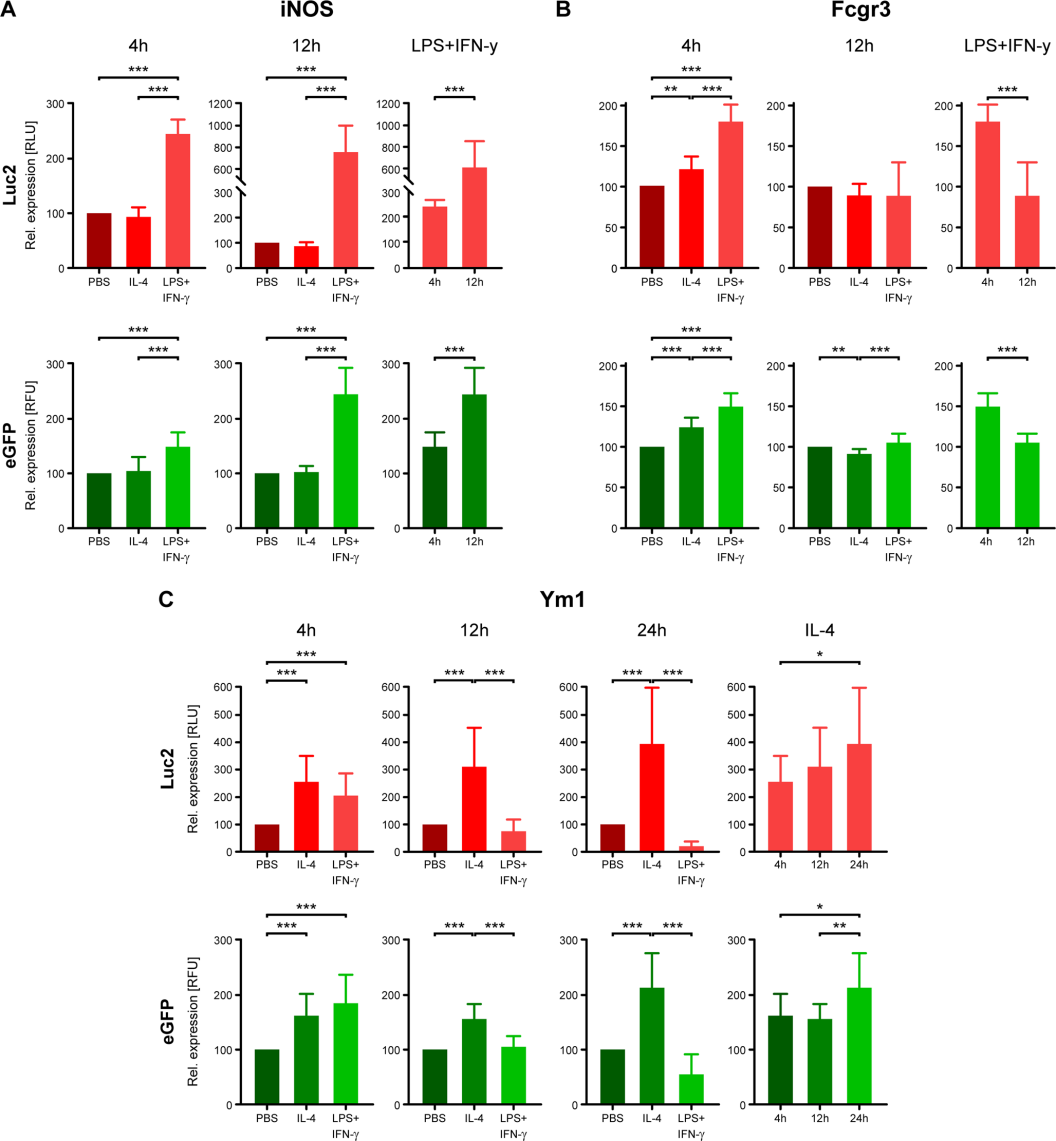
**Reporter protein activity after stimulation of BV-2 cells transduced with the polarisation reflecting promoters. a**, **b** M1-like iNOS and Fcgr3 promoters were activated by LPS + IFN-γ with a significant increase after 4 h and 12 h, and after 4 h, respectively. **c** M2-like Ym1 promoter was significantly activated by IL-4 stimulation, but down-regulated by LPS + IFN-γ at 12 h and 24 h. Luc2 and eGFP levels were measured using a Mithras^2^ LB943 plate reader (*n* = 6 wells; 3 independent experiments for each group). Signal intensity values of stimulated conditions were expressed in relative units to PBS condition, set to 100%. Mean ± standard deviation is shown. * *p* ≤ 0.05, ** *p* ≤ 0.01, *** *p* ≤ 0.001

In BV-2 iNOS-Luc2-T2A-eGFP cells, both Luc2 and eGFP signal intensities significantly increased after 4 h (*p* < 0.001 for Luc2 RLU and eGFP RFU) and 12 h (*p* < 0.001 for RLU and RFU) of LPS + IFN-γ stimulation compared to PBS and IL-4 conditioned cells (Fig. 4a). Here, RLU and RFU reached statistically significantly higher levels after 12 h compared to 4 h (p < 0.001). As expected, neither RLU nor RFU increased after IL-4 treatment at any time point. This behaviour was fully in line with the WB findings (Fig. 2): iNOS expression levels and RLU/RFU activity were increased after 12 h compared to 4 h of LPS + IFN-γ stimulation. Compared to PBS treated cells, IL-4 treatment did not change reporter activity or iNOS protein levels at any time point.

Stimulated BV-2 Fcgr3-Luc2-T2A-eGFP cells showed distinct activation patterns of reporters compared to protein levels of WB lysates. Here, contrary to WB results, RLU and RFU significantly increased after 4 h of both, LPS + IFN-γ (*p* < 0.001 for RLU and RFU) and IL-4 stimulation (*p* = 0.001 for RLU; p < 0.001 for RFU), compared to PBS conditioned cells (Fig. 4b). Even more, Luc2 and eGFP activities were significantly increased after LPS + IFN-γ compared to IL-4 stimulation (*p* < 0.001 for both). This increase after 4 h of either stimulation condition was not observed in WB analysis. Interestingly, after 12 h this increase vanished for both stimulation cues, and upon IL-4 stimulation, RLU and RFU even slightly decreased below the baseline level of PBS treated cells, with a statistically significant decrease for eGFP (*p* = 0.006). eGFP activity was still significantly higher in LPS + IFN-γ stimulated cells compared to IL-4 treated cells (*p* < 0.001). These findings differ in part from the WB analysis for the Fcgr3 behaviour after stimulation. While Fcgr3 protein levels of lysates increased with longer IL-4 stimulation, reporter activity decreased again. However, the decrease upon LPS + IFN-γ stimulation in both RLU and RFU after 12 h compared to 4 h (p < 0.001), reflecting a reduction in promoter activity over time, is in complete agreement with down-regulated Fcgr3 protein levels found in lysates over time (Fig. 2).

In BV-2 Ym1-Luc2-T2A-eGFP cells, bioluminescence and fluorescence signals significantly increased upon IL-4 stimulation at all three time points, compared to PBS conditioned cells (*p* < 0.001 for 4 h, p < 0.001 for 12 h, p < 0.001 for 24 h RLU; *p* = 0.001 for 4 h, p < 0.001 for 12 h, p < 0.001 for 24 h RFU) (Fig. 4c). The strongest increase of reporter protein activity upon IL-4 stimulation was observed after 24 h (with *p* = 0.02 between 4 and 24 h and *p* = 0.008 between 12 and 24 h for RFU; *p* = 0.041 between 4 and 24 h for RLU). After 4 h of LPS + IFN-γ stimulation, however, both RLU und RFU also significantly increased, compared to PBS condition (*p* = 0.01 for RLU; p < 0.001 for RFU). Here, RLU increased to a lesser, and RFU to a higher extent than in IL-4 stimulated cells. These significant increases of RFU and RLU upon IL-4 and LPS + IFN-γ stimulation after 4 h were not observed on protein levels analysed by WB. After 12 and 24 h, LPS + IFN-γ stimulation resulted in significantly lower RLU and RFU compared to IL-4 stimulation (p < 0.001 for 12 h, p < 0.001 for 24 h RLU; p < 0.001 for 12 h, p < 0.001 for 24 h RFU) with levels similar to PBS stimulation after 12 h. After 24 h, LPS + IFN-γ stimulation resulted in a clear opposite effect than IL-4 treatment, as both, RLU and RFU, decreased compared to PBS condition (*p* = 0.058). In summary, the continuous reporter increase upon IL-4 stimulation over time and significantly lower levels upon LPS + IFN-γ stimulation compared with IL-4 stimulated cells are in line with the WB data. Further, the late response of Ym1 after IL-4 stimulation is in line with earlier reports in the literature (Menzies et al. 2010).

## Discussion

Here, we demonstrate an imaging reporter strategy to follow the polarisation states of microglial cells under specific stimulation conditions. For this purpose, we established stable BV-2 cell lines through stable integration of the reporter genes Luc2 and eGFP controlled by the M1- or M2-like phenotype-representing iNOS-, Fcgr3- and Ym1-promoters in the microglial cell genome by lentiviral transduction. The promoter activity was then monitored by reporter gene activity following appropriate stimulations. eGFP expression was first assessed under the microscope, while quantitative measures of Luc2 and eGFP activity by plate reader, measured in RLU and RFU, respectively, allowed quantitative analysis of promoter activity. Here, through the T2A peptide, Luc2 and eGFP are co-expressed at equivalent levels (de Felipe et al. 2006).

Before validating the reporter system, we first tested vitality of BV-2 cells during the various stimulation conditions. There was no statistically significant difference in vitality between naïve and transduced cells and also no significant difference between unstimulated and stimulated cells. Only in the case of LPS + INFγ stimulation for long durations of 24 h a substantial vitality decrease was noted. In this case, additional influence of the dead cells in the vicinity on the observed stimulation profile cannot be fully excluded, and thus results of long duration LPS + INFγ stimulation should be interpreted with caution. However, dead cells would most likely stimulate microglia to become of pro-inflammatory phenotype, thus only further enhancing the direction already induced by the LPS + INFγ stimulus. We then analyzed stimulation conditions on BV-2 wt cells by WB, and assessed well-known inflammatory markers based on literature (Martinez and Gordon 2014; Jablonski et al. 2015; Orihuela et al. 2016). In order to confirm that lentiviral transduction does not influence phenotype marker expression, we repeated stimulation assays in BV-2 cells which had been stably transduced with LV-EF1α-Luc2-T2A-eGFP and sorted by FACS. We chose iNOS, Fcgr3 and Ym1 to study promoter activity, since these genes are well known for their responses to inflammatory stimuli (Chang et al. 2001; Hu et al. 2012; Hamzei Taj et al. 2016). As pro-inflammatory stimulus driving the microglia into an M1-like phenotype, the strong combination of LPS and IFN-γ was applied. For the induction of M2-like, anti-inflammatory phenotype, the cytokine IL-4 was used.

### Protein Expression after Stimulation of BV-2 Microglia

We found that

1. iNOS was exclusively expressed in cells stimulated with LPS + IFN-γ at all time points of 4, 21 and 30 h.
2. Fcgr3 expression levels were higher in IL-4 stimulated cells compared to LPS + IFN-γ treated cells.
3. Ym1 was strongly up-regulated upon IL-4 at 21 and 30 h and not expressed upon LPS + IFN-γ stimulation.

The observation of the same expression patterns in BV-2 EF1α-Luc2-T2A-eGFP cells as in wt BV-2 cells demonstrated convincingly that cell transduction had no artefactual stimulating effect on the BV-2 microglia cell line, thus accessing the study of reporter gene activity in transduced cells.

### Methodological Considerations for RFU and RLU Behaviour

The co-expression of Luc2 and eGFP at closely identical levels allows for bivalent information and control. However, we found some differences between RLU and RFU among the same stimulated cell populations. Firefly luciferase can convert its required substrate luciferin solely in the presence of ATP (de Wet et al. 1987), and therefore emits photons in living cells only. Additionally, there is no auto-luminescence background while auto-fluorescence must be considered for analysis of fluorescence data. eGFP is not only prone to photo bleaching, it also needs long time to mature and has a long lifetime (Fan and Wood 2007). The stability of reporter proteins influences the inference of the transcription rate (Allard 2008). Thus, eGFP concentration changes only slowly upon transcriptional alterations, and hence, veils detection of promoter activity and subsequent biological changes. In order to track concentration changes faster upon transcriptional regulation, reporters could in future experiments be fused with protein degradation sequences (Allard 2008). In summary, lower RFU compared to RLU values can be explained by slower eGFP concentration change or can be due to background correction due to auto-fluorescence of the BV-2 wt control cells. Additionally, even though we counted cells and transferred equal numbers of living cells into test wells, we cannot rule out the presence of dead cells as well, especially after longer stimulation. The fact that dead cells show higher auto-fluorescence could be another reason for the observed discrepancy and, in summary, point to a higher reliability with a 10–1000 fold higher sensitivity rate of quantitative results by bioluminescence than by fluorescence (Allard 2008).

### Agreement/Disagreement between Protein Expression and Gene Activity Monitoring

While essentially there is very good agreement between WB results and reporter signal behaviour upon stimulation, we found some discrepancies between WB experiments and plate reader analysis for Fcgr3 protein levels and promoter activity. While WB provides information about protein levels, the plate reader measurements allow sensitive monitoring of the early state of promoter activity response on the gene level. Hence, the reporter strategy focuses on the promoter activity, while it is not sensitive to any post-transcriptional or post-translational modifications, and possible interactions through signalling pathways are not taken into account. Furthermore, mRNA levels can be influenced by various modulatory factors such as e.g. siRNA and miRNA, resulting in altered protein levels (Decker and Parker 2012). Discrepancies of our reporter based results to the WB results may be explained only partially by methodological aspects: the promoter sequence chosen, and thus response elements and recognition sites for transcription factors, might differ to some extent from the naïve promoter regions. With the Basic Local Alignment Search Tool (BLAST) we assured 99% identity of our promoter sequence to the murine Fcgr3 promoter region (accession number U11854), also consisting of, based on (Feinman et al. 1994), the two *cis*-acting elements PU.1 and MyE, necessary for proper promoter function. Further, for WB we chose a polyclonal antibody, thus binding to various epitopes, which could cause less specificity. Fcgr3 and Fcgr2 share 95% of the same amino acid sequences of the extracellular domain (Ravetch and Kinet 1991) and reveal a similar molecular weight of 35–40 kDA. WB analysis of Fcgr2 (data not shown) resulted in an equivalent protein up-regulation upon IL-4, but not upon LPS + IFN-γ. This is in line with reports by Nimmerjahn and Ravetch, showing an Fcgr2 expression increase upon IL-4 and a down-regulation upon IFN-γ stimulation (Nimmerjahn and Ravetch 2006).

Weinshank et al. reported that IFN-γ stimulation results in an increase of both, Fcgr3 mRNA and protein levels, in macrophage RAW 264.7 and J774.A cells (Weinshank et al. 1988), in contradiction to our observation of low Fcgr3 protein levels after LPS + IFN-γ stimulation. Reasons for this difference may be the different cell types (macrophages in Weinshank studies vs. microglia in our approach). Further reasons may lie in the specific experimental conditions which in our case inhibited Fcgr3 translation by unknown reasons. The observed increase after IL-4 stimulation (at 21 h and 30 h with WB, and at 4 h with plate reader analysis) is in line with Chhor et al., who reported up-regulated CD16 mRNA levels upon IL-4 stimulation in primary murine microglia, peaking at 4 h and decreasing thereafter (Chhor et al. 2013). However, this report is contradictory to the findings by Chauhan and colleagues, who did not find any change of Fcgr3, but an increase of Fcgr2b mRNA levels upon IL-4 at 6 and 24 h in primary murine microglia (Chauhan et al. 2017). This example shows that the role of Fcgr3 in microglia polarisation response is still greatly unclear in the literature. Further, even though activation of Fc gamma receptors is reported to cause generation of pro-inflammatory cytokines and chemokines, activation in the presence of Toll-like receptors (TLR) activated by their ligands, such as IFN-γ, induces a so-called M2b – regulatory phenotype (Guilliams et al. 2014).

The increase of reporter activity after 4 h of LPS + IFN-γ stimulation in microglia transduced with the Ym1 promoter might unravel an early effect on transcriptional regulation, which has not been reported in the literature so far. Alternatively, this effect could be due to the uniqueness of combination and applied concentration of stimulation agents together with the BV-2 cell line. However, future experiments are required to unequivocally analyse the underlying reasons for the observation of early Ym1 signal increase after LPS + IFN-γ stimulation.

Taken together, discrepancies in the literature not only exist for Fcgr3 response upon stimulation. The varying use of stimulating concentrations, the type of agents, cells, and stimulation timing or experimental methods differ in such a wide range, that overall conclusions are still restricted.

### Perspectives of the Novel Strategy of Monitoring Polarisation Changes by Reporter Gene Imaging

The induction of inflammatory responses is a highly complex process and involves multiple factors and signalling pathways. Here, we have developed a tool focusing on the study of genes involved in the inflammatory processes. We are able to monitor promoter activity in the early state of response to stimulation in cell culture by highly sensitive reporter imaging. Compared to methods of protein detection, our method provides access to understanding early regulation before protein expression takes place. Hence, this approach looks at the very early step of cell response to a stimulus. It also facilitates screening of new drugs of immune modulatory treatment strategies while further selection of genes for reporter imaging identifies underlying mechanisms. Finally, it can be envisioned that intracerebral injection of these LV (or AAV) with the polarization reporters in animals will generate time profiles of polarization changes under spontaneous or drug-modulated inflammatory response.

## Conclusions

Our new imaging reporter strategy permits easy and rapid monitoring of microglia polarisation changes upon stimulation by various activators such as cytokines or bacterial activators (LPS). The presented method opens new doors for deciphering the role of the anti- and pro-inflammatory polarisation phases of brain diseases. Furthermore, it allows the controlled screening of drugs influencing the immune response by modulating the polarisation states, thus leading to a better understanding of the gene activities involved.

## Electronic supplementary material

ESM 1 (DOCX 15 kb)

Suppl. Fig. 1. **pGL2-NOS2Promoter-Luciferase (Addgene plasmid #19296).** NIH accession number L09126.1 (GIF 55 kb)

High Resolution Image (TIF 4234 kb)

Suppl. Fig. 2. Fcgr3 (MPRM15197-PG04, Genecopoeia). (GIF 72 kb)

High Resolution Image (TIF 4770 kb)

Suppl. Fig. 3. **Ym1 (MPRM18306-PG04, Genecopoeia).** NIH accession number NM_009892 (GIF 61 kb)

High Resolution Image (TIF 4548 kb)

Suppl. Fig. 4. pCDH-EF1α-Luc2-T2A-eGFP. (GIF 59 kb)

High Resolution Image (TIF 4236 kb)

Suppl. Fig. 5. pCDH-iNOS-Luc2-T2A-eGFP. (GIF 55 kb)

High Resolution Image (TIF 3976 kb)

Suppl. Fig. 6. pCDH-Fcgr3-Luc2-T2A-eGFP. (GIF 60 kb)

High Resolution Image (TIF 4361 kb)

Suppl. Fig. 7. pCDH-Ym1-Luc2-T2A-eGFP. (GIF 60 kb)

High Resolution Image (TIF 4050 kb)

Suppl. Fig. 8. **FACS of transduced BV-2 cells.** BV-2 EF1-Luc2-T2A-eGFP sorted. BV-2 iNOS-Luc2-T2A-eGFP, high eGFP sorted. BV-2 iNOS-Luc2-T2A-eGFP, middle eGFP sorted. BV-2 Fcgr3-Luc2-T2A-eGFP sorted. BV-2 Ym1-Luc2-T2A-eGFP sorted (GIF 116 kb)

High Resolution Image (TIF 8066 kb)

Suppl. Fig. 9. **Vitality of naïve and transduced microglia BV-2 under stimulated conditions.** Vitality of microglia was assessed using a Countess automated cell counter and expressed in percent of number of cells analyzed. Comparison of the three transgenic cell lines (BV-Fcgr3, BV-iNOS,and BV-Ym1) with wild type cells of same condition is presented. Statistical analysis showed no difference between transduced and naïve cells. Also, no influence of stimulation condition on vitality was observed with the exception of LPS + INFγ stimulation for long stimulation periods of 24 h. (GIF 101 kb)

High Resolution Image (TIF 6378 kb)

Suppl. Fig. 10. **Sorted BV-2 EF1α-Luc2-T2A-eGPF cells in comparison with BV-2 wt cells.** Overlays of BV-2 EF1α-Luc2-T2A-eGFP cells 2 days after FACS on top: Cells were sorted based on middle (left) and high eGFP expression (right). 20X magnification. For comparison, BV-2 wt cells below. 10X magnification left, scale bar 100 μm. 20X magnification right, scale bar 50 μm. (GIF 122 kb)

High Resolution Image (TIF 9623 kb)
