# Supplementary material for: Imaging Reporter Strategy to Monitor Gene Activation of Microglia Polarisation States under Stimulation
Source: J Neuroimmune Pharmacol. 2018 May 22;13(3):371–82. doi: 10.1007/s11481-018-9789-2 (PMC6096558; doi:10.1007/s11481-018-9789-2)
Supplement: Supplementary file 1 — (DOCX 15 kb) [file 11481_2018_9789_MOESM1_ESM.docx]

**S Table 1. Primer sequences for cloning of promoters.**

| Promoter | Cloning primer 5´→3´ |
| --- | --- |
| Ym1_ClaI_for | AAGGGAAAAA ATCGAT GGGGATAGGAGC |
| Ym1_XbaI_rev | TAAAAGCG TCTAGA TGGTGTCTTCAGG |
| iNOS_ClaI_for | AAGGGAAAAA GCCACC ATCGAT GACTTTGATATG |
| iNOS_BamHI_rev | TAAAAGCG GGATCC GACTAGGCTACTC |
| Fcgr3_ClaI_for | AAGGGAAAAA ATCGAT TCTGCCACTGTGAACC |
| Fcgr3_XbaI_rev | TAAAAGGCG TCTAGA TCTGGGTGTCCAAAGTC |

**S Table 2. Western Blot antibody list.**

**Primary antibodies**

|  | Supplier | Catalog number | kDA | Concentration |
| --- | --- | --- | --- | --- |
| rb α-iNOS | Cell Signaling Technology, Cambridge, UK | D6B6S | 130 | 1:1,000 |
| go α-Fcgr3 | R&D Systems GmbH, Wiesbaden, Germany | AF1960 | 35-40 | 1:2,500 |
| rb α-Ym1 | Abcam, Cambridge, UK | Ab93034 | 44 | 1:500 |
| mo α-CD206 | R&D Systems GmbH, Wiesbaden, Germany | MAC25341 | 150 | 1:200 |
| rb α-Arginase 1 | Genetex Inc., Irvine, CA, USA | GTX109242 | 35 | 1:5,000 |
| mo α-Actin | MP Biomedicals, Santa Ana, CA, USA | 69100 | 42 | 1:5,000 |

**Secondary antibodies, IgG HRP conjugates**

|  | Supplier | Catalog number | Concentration |
| --- | --- | --- | --- |
| go α-mouse | EMD Millipore, Billerica, MA, USA | 12-349 | 1:3,000 |
| go α-rabbit | Cell signaling Technology, Cambridge, UK | 7074 | 1:800 |
| do α-goat | Santa Cruz Biotechnology, Dallas, TX, USA | sc-2020 | 1:5,000 |

Abbreviations: Do, donkey; go, goat; mo, mouse; rb, rabbit.
