# Supplementary figures and images for: Imaging Reporter Strategy to Monitor Gene Activation of Microglia Polarisation States under Stimulation
Source: J Neuroimmune Pharmacol. 2018 May 22;13(3):371–82. doi: 10.1007/s11481-018-9789-2 (PMC6096558; doi:10.1007/s11481-018-9789-2)

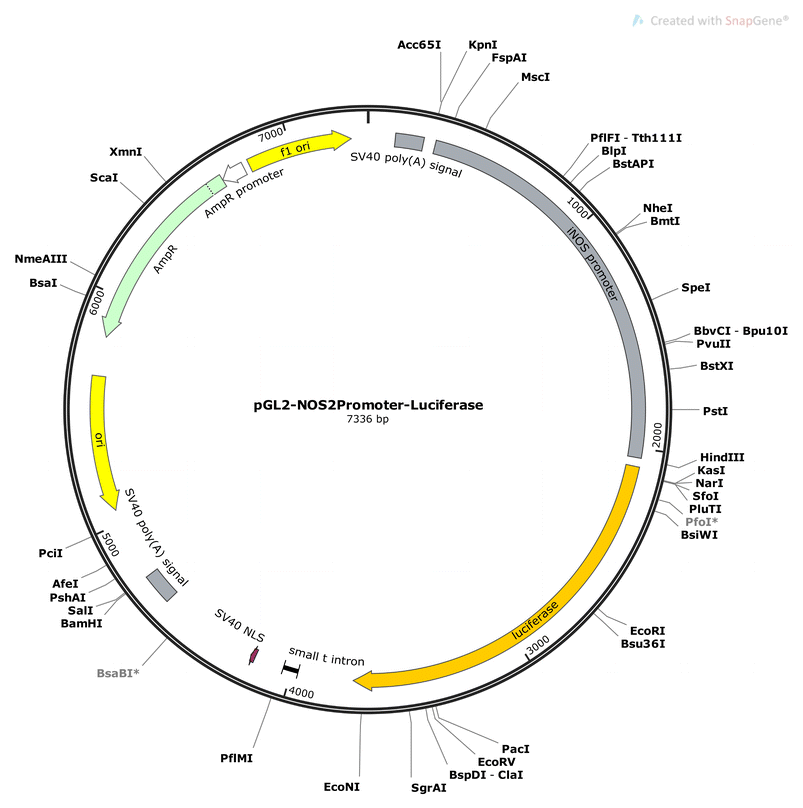

Supplement: Supplementary file 2 — pGL2-NOS2Promoter-Luciferase (Addgene plasmid #19296). NIH accession number L09126.1 (GIF 55 kb) [file 11481_2018_9789_Fig5_ESM.gif]

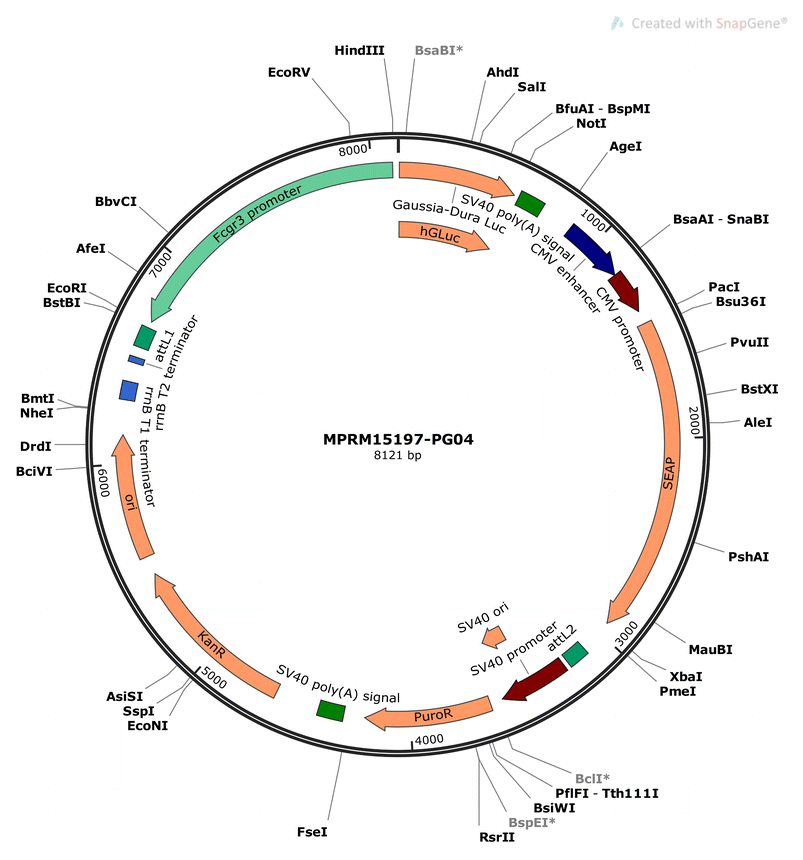

Supplement: Supplementary file 4 — Fcgr3 (MPRM15197-PG04, Genecopoeia). (GIF 72 kb) [file 11481_2018_9789_Fig6_ESM.gif]

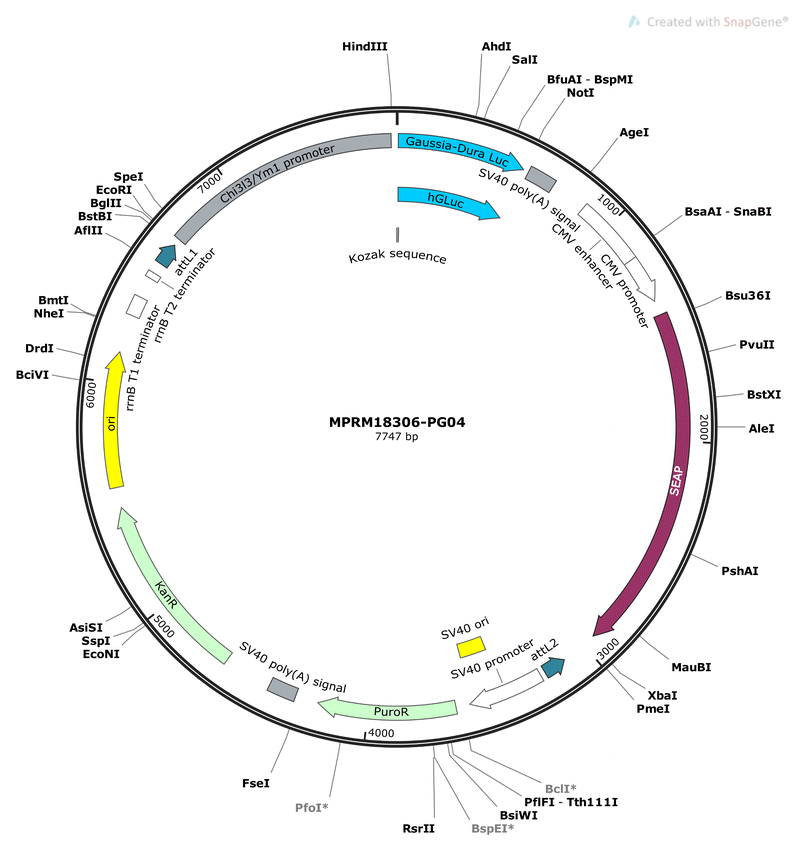

Supplement: Supplementary file 6 — Ym1 (MPRM18306-PG04, Genecopoeia). NIH accession number NM_009892 (GIF 61 kb) [file 11481_2018_9789_Fig7_ESM.gif]

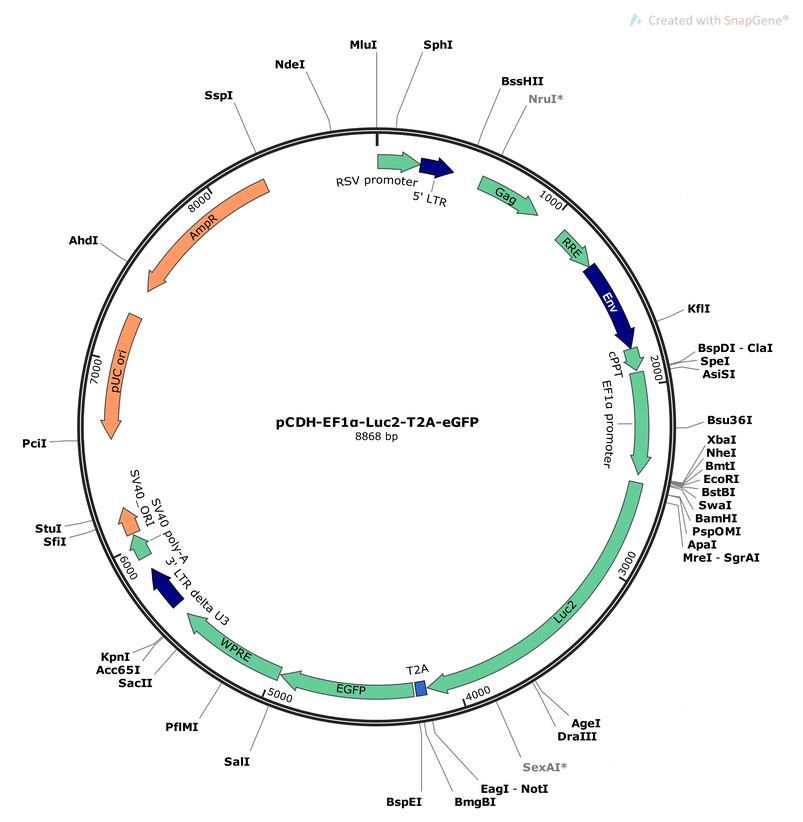

Supplement: Supplementary file 8 — pCDH-EF1α-Luc2-T2A-eGFP. (GIF 59 kb) [file 11481_2018_9789_Fig8_ESM.gif]

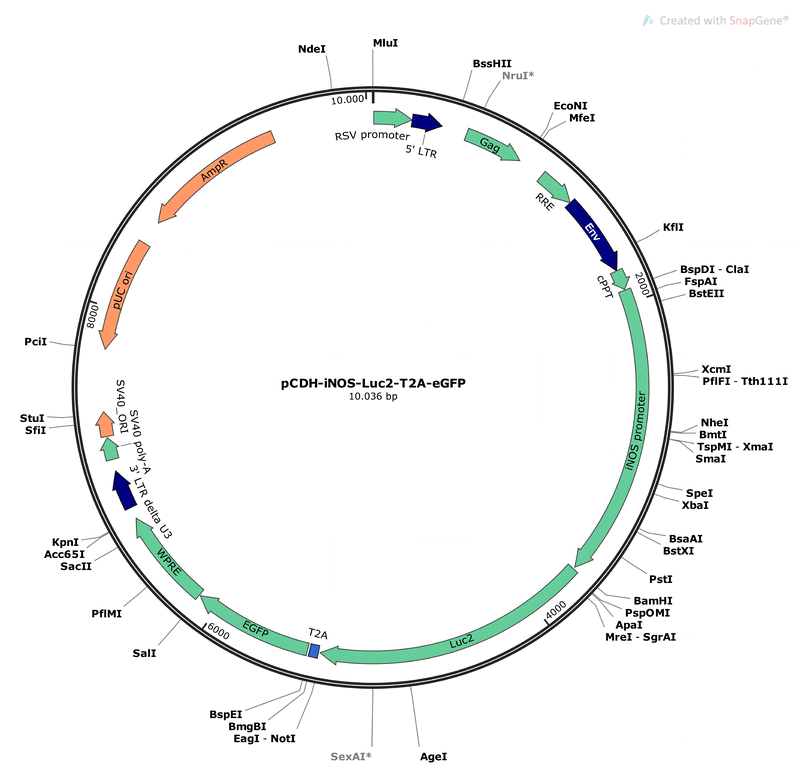

Supplement: Supplementary file 10 — pCDH-iNOS-Luc2-T2A-eGFP. (GIF 55 kb) [file 11481_2018_9789_Fig9_ESM.gif]

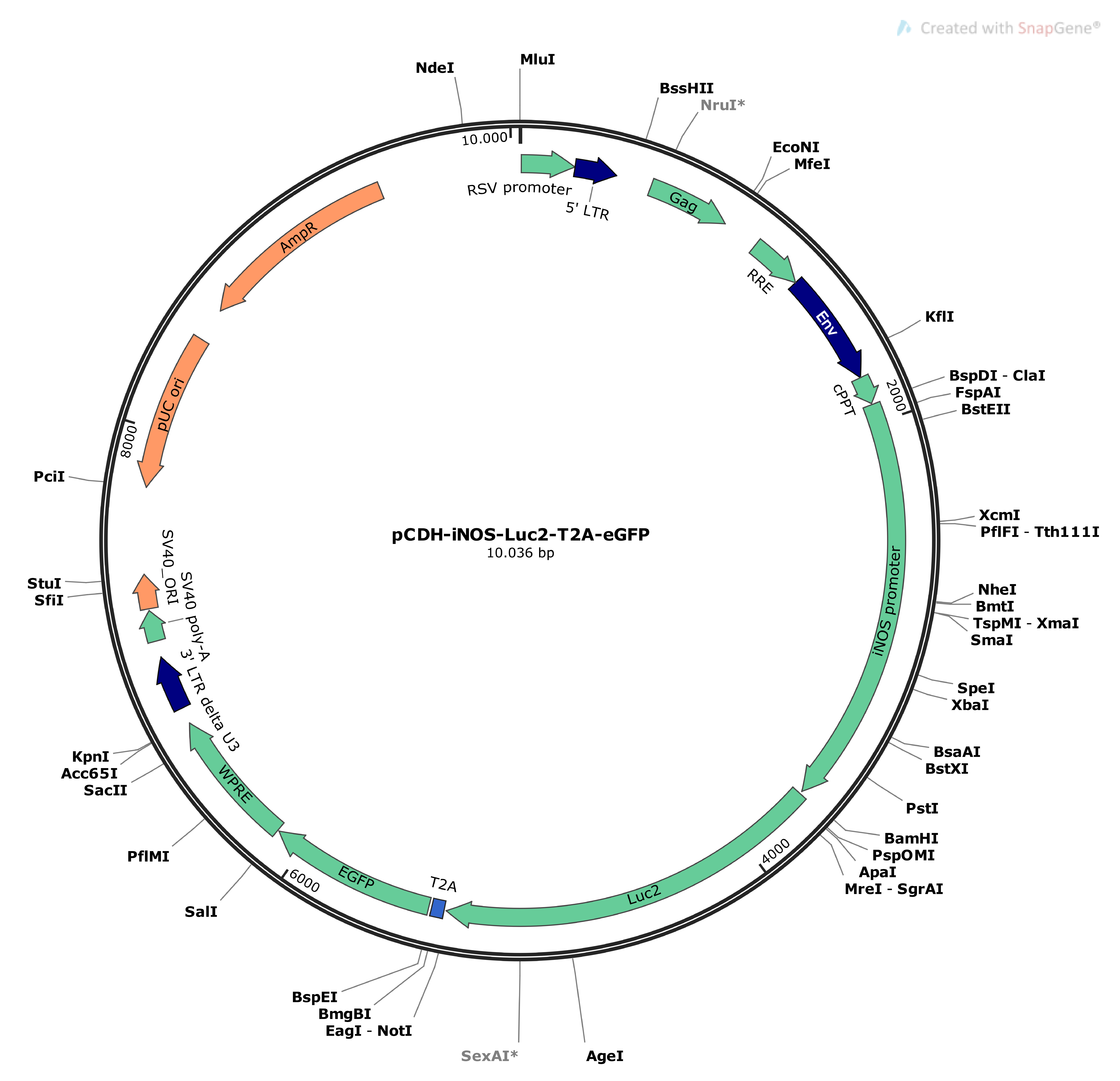

Supplement: Supplementary file 11 — High Resolution Image (TIF 3976 kb) [file 11481_2018_9789_MOESM6_ESM.tif]

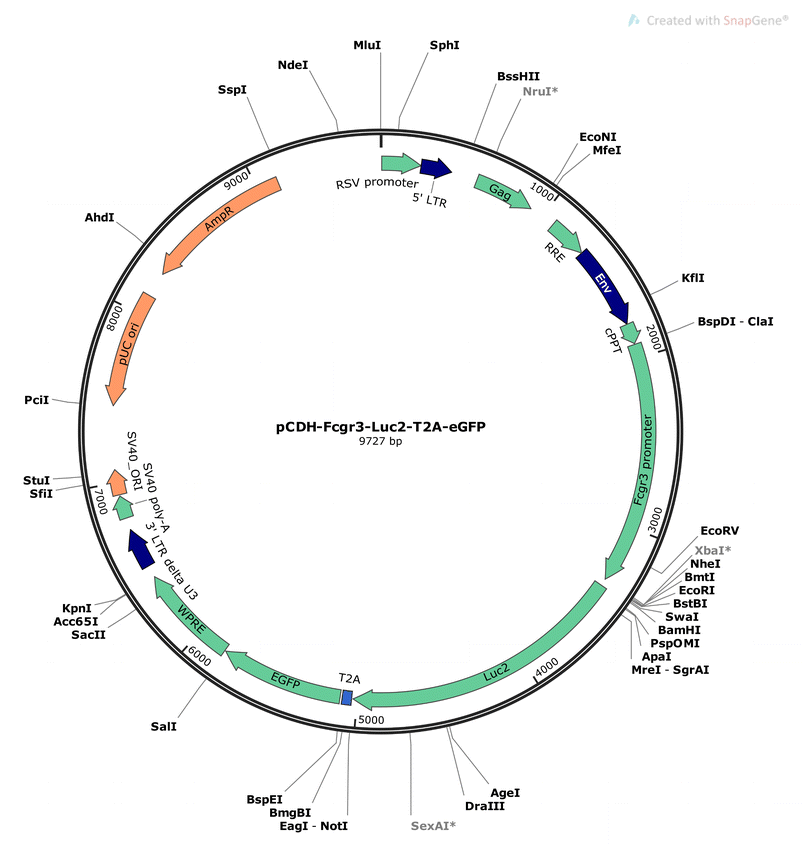

Supplement: Supplementary file 12 — pCDH-Fcgr3-Luc2-T2A-eGFP. (GIF 60 kb) [file 11481_2018_9789_Fig10_ESM.gif]

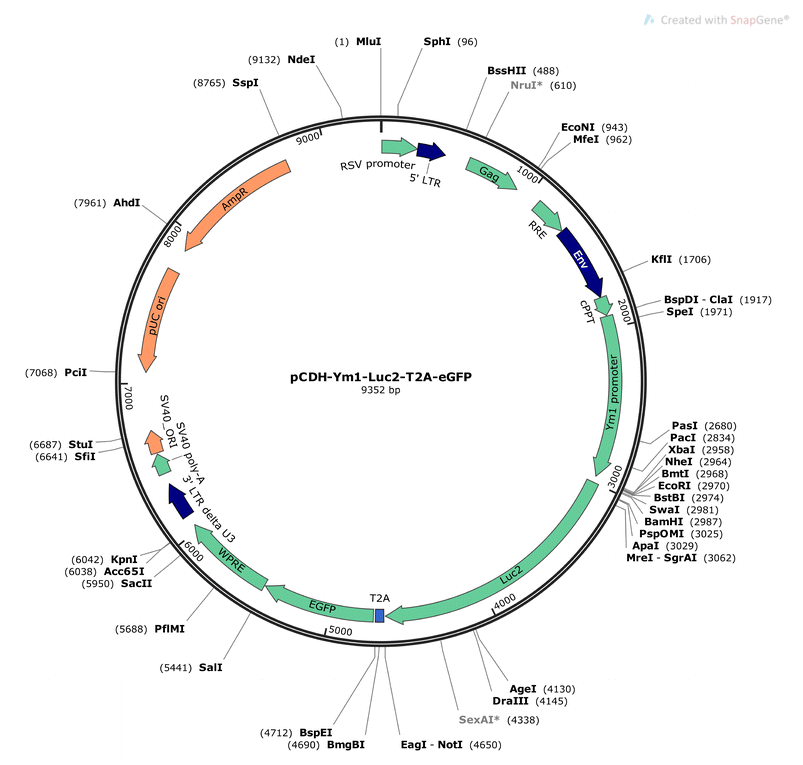

Supplement: Supplementary file 14 — pCDH-Ym1-Luc2-T2A-eGFP. (GIF 60 kb) [file 11481_2018_9789_Fig11_ESM.gif]

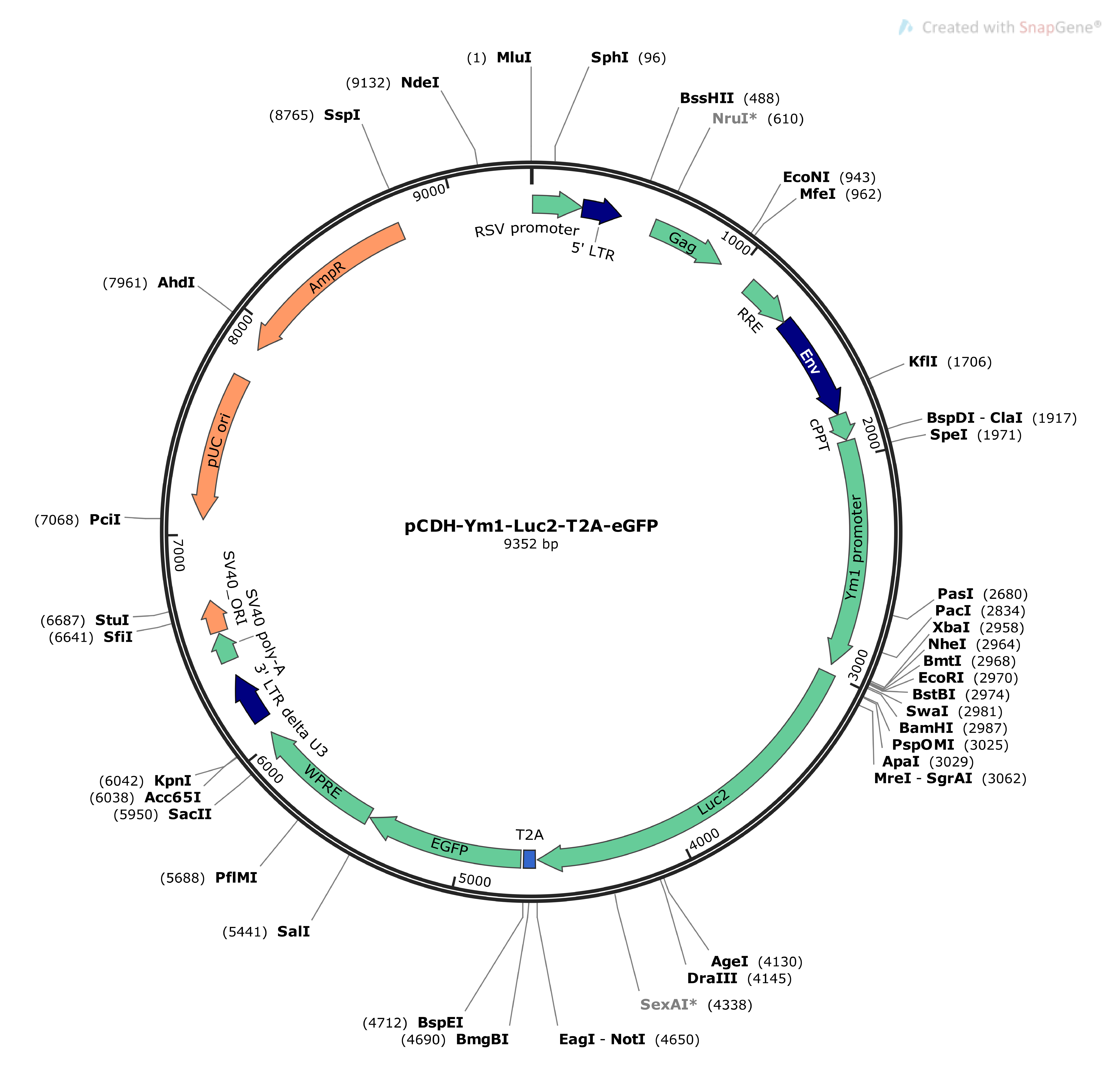

Supplement: Supplementary file 15 — High Resolution Image (TIF 4050 kb) [file 11481_2018_9789_MOESM8_ESM.tif]

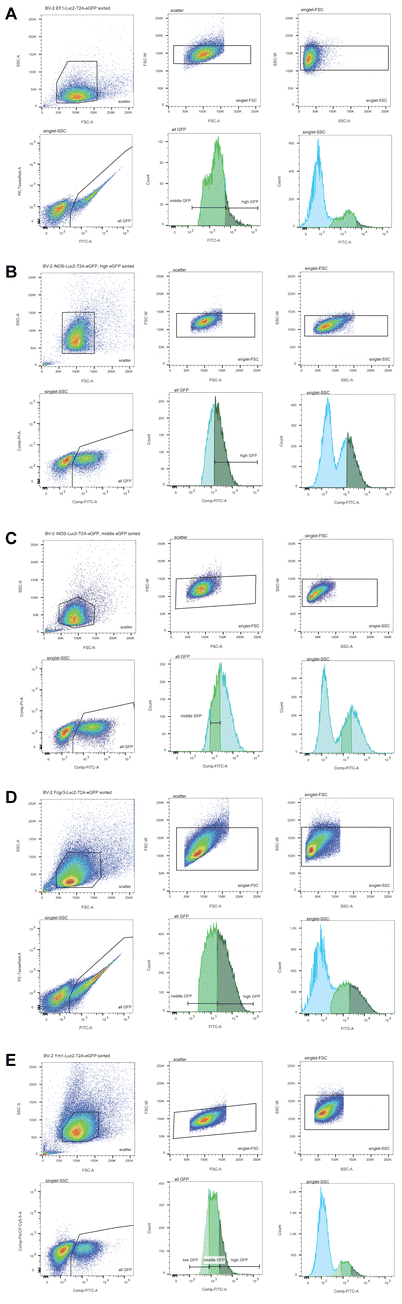

Supplement: Supplementary file 16 — FACS of transduced BV-2 cells. BV-2 EF1-Luc2-T2A-eGFP sorted. BV-2 iNOS-Luc2-T2A-eGFP, high eGFP sorted. BV-2 iNOS-Luc2-T2A-eGFP, middle eGFP sorted. BV-2 Fcgr3-Luc2-T2A-eGFP sorted. BV-2 Ym1-Luc2-T2A-eGFP sorted (GIF 116 kb) [file 11481_2018_9789_Fig12_ESM.gif]

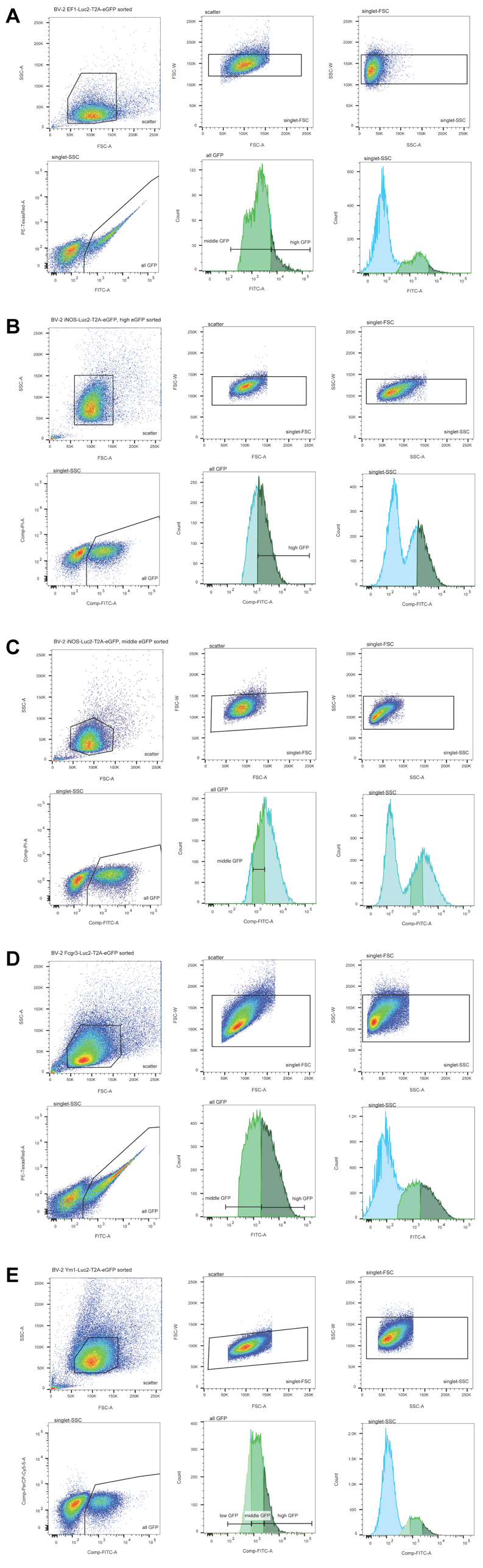

Supplement: Supplementary file 17 — High Resolution Image (TIF 8066 kb) [file 11481_2018_9789_MOESM9_ESM.tif]

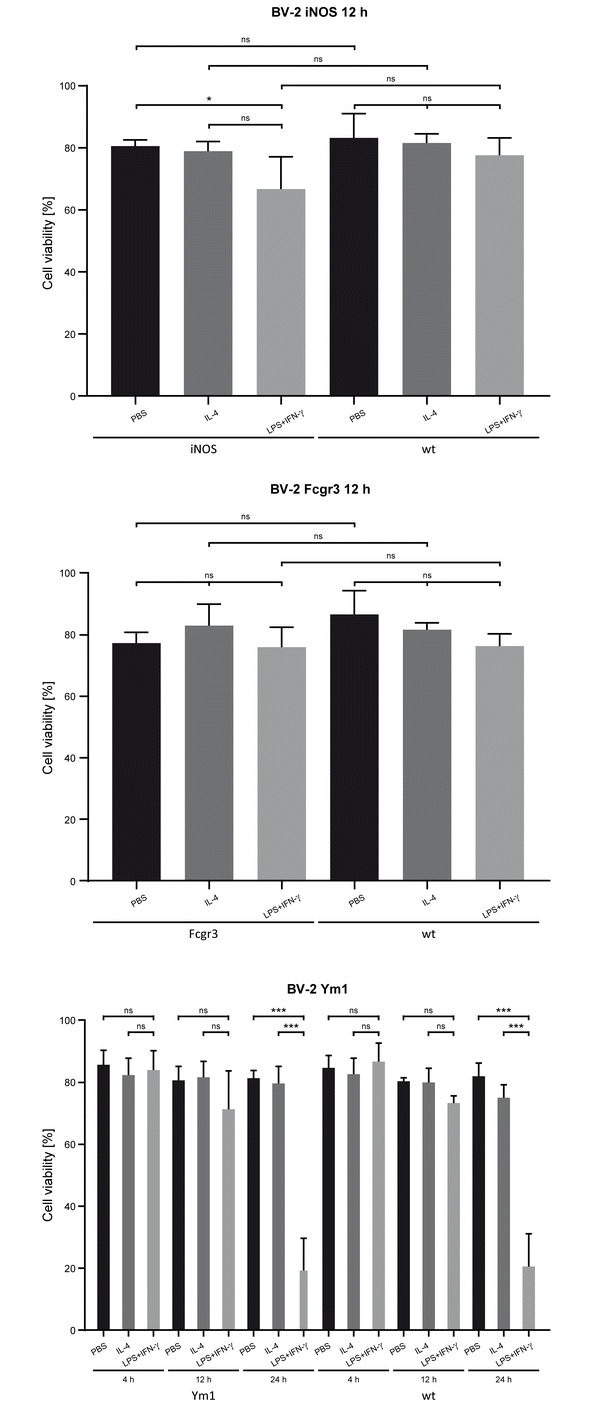

Supplement: Supplementary file 18 — Vitality of naïve and transduced microglia BV-2 under stimulated conditions. Vitality of microglia was assessed using a Countess automated cell counter and expressed in percent of number of cells analyzed. Comparison of the three transgenic cell lines (BV-Fcgr3, BV-iNOS,and BV-Ym1) with wild type cells of same condition is presented. Statistical analysis showed no difference between transduced and naïve cells. Also, no influence of stimulation condition on vitality was observed with the exception of LPS + INFγ stimulation for long stimulation periods of 24 h. (GIF 101 kb) [file 11481_2018_9789_Fig13_ESM.gif]

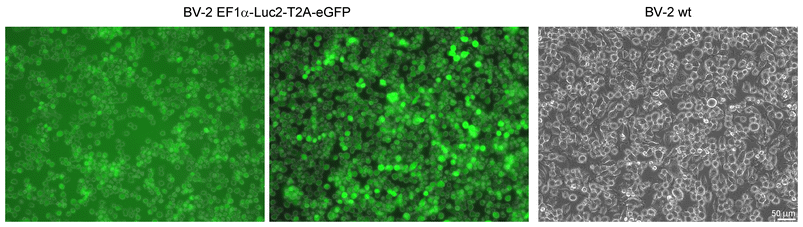

Supplement: Supplementary file 20 — Sorted BV-2 EF1α-Luc2-T2A-eGPF cells in comparison with BV-2 wt cells. Overlays of BV-2 EF1α-Luc2-T2A-eGFP cells 2 days after FACS on top: Cells were sorted based on middle (left) and high eGFP expression (right). 20X magnification. For comparison, BV-2 wt cells below. 10X magnification left, scale bar 100 μm. 20X magnification right, scale bar 50 μm. (GIF 122 kb) [file 11481_2018_9789_Fig14_ESM.gif]

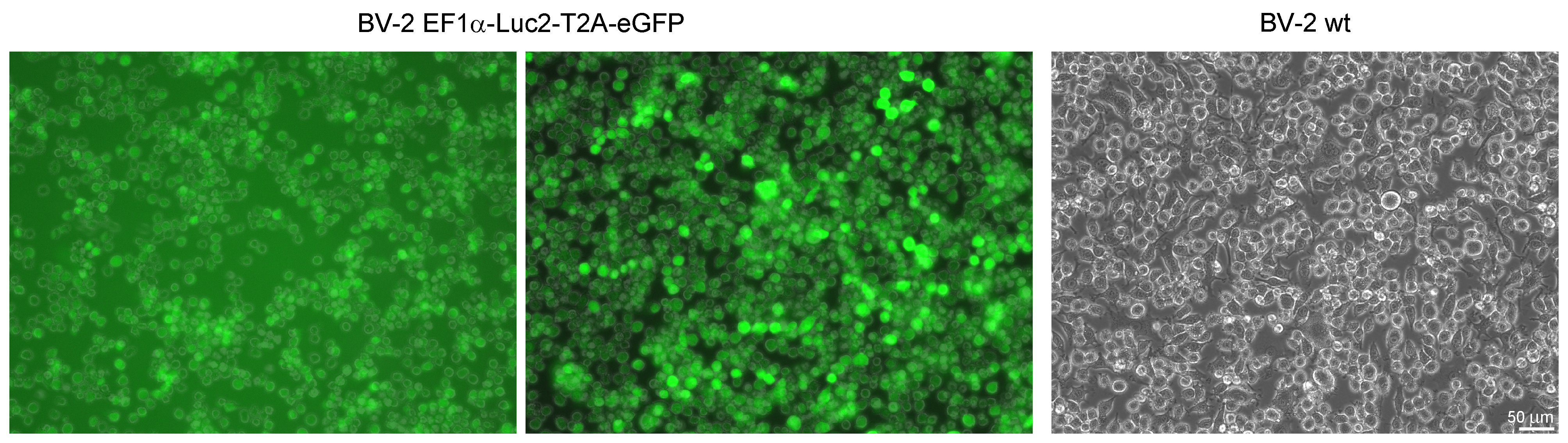

Supplement: Supplementary file 21 — High Resolution Image (TIF 9623 kb) [file 11481_2018_9789_MOESM11_ESM.tif]
